# Pit Latrines and Groundwater Contamination: Negative Impacts of a Popular Sanitation Method

**DOI:** 10.1289/ehp.121-a169

**Published:** 2013-05-01

**Authors:** Tanya Tillett

**Affiliations:** Tanya Tillett, MA, of Durham, NC, is a staff writer/editor for *EHP*. She has been on the *EHP* staff since 2000 and has represented the journal at national and international conferences.

Worldwide an estimated 1.77 billion people use pit latrines as their primary mode of sanitation. With the prevalence of pit latrine use growing in developing countries, there is heightened concern about the negative impacts on drinking water. A new review assesses the known and measured environmental health impacts associated with groundwater contamination by pit latrines [*EHP* 121(5):521–530; http://dx.doi.org/10.1289/ehp.1206028].

By 2015 the United Nations aims to increase by nearly 2 billion the number of people with sustainable access to “improved” sanitation. “Improved” means the waste is kept separate from the people who use the facilities. Covered pit latrines, although basic, are a vast improvement over open defecation and other unsanitary forms of waste management. But because pit latrines usually lack a physical barrier to keep waste contained in the pit, microbial and chemical contaminants can emanate from them, threatening nearby groundwater sources such as springs, wells, and boreholes.

The authors of the current review used survey data from the U.S. Agency for International Development, the World Health Organization, and the United Nations Children’s Fund to calculate global use of improved (covered with a platform or concrete slab) and unimproved (no platform or slab) sanitation units; excreta production; and groundwater use. The team also conducted a literature search for groundwater contamination associated with pit latrines.

**Figure:**
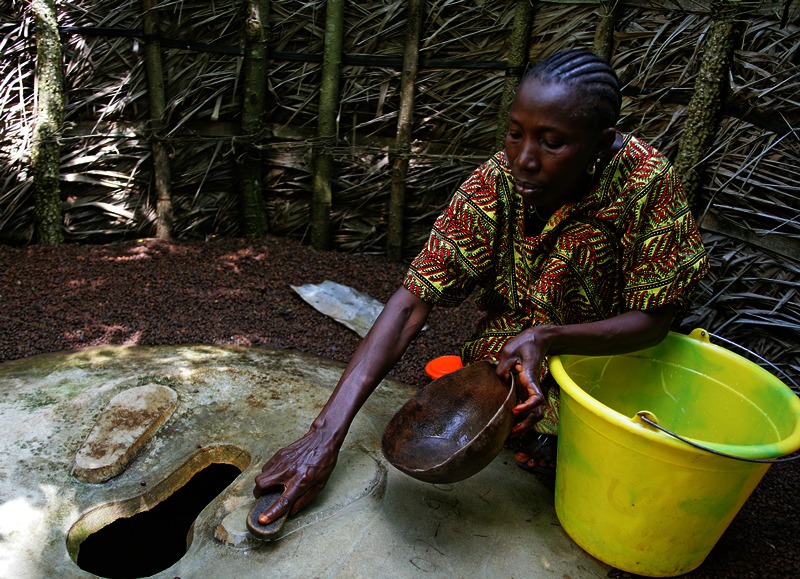
A woman cleans an improved pit latrine in Guinea. Two-thirds of households in Guinea use pit latrines as their primary method of sanitation, and nearly that many rely on groundwater for their drinking water. © Giacomo Pirozzi / Panos Pictures

In some countries of West Africa and the Middle East, the vast majority of the population uses pit latrines. Countries where pit latrine use is prevalent also tend to have high rates of groundwater use. Siting recommendations appeared to vary across countries, ranging anywhere from 15 to 75 meters between water sources and sanitation units.

Studies have associated pit latrine use with the transport of microbes (typically fecal coliforms, although one study assessed adenovirus and rotavirus) and chemicals (e.g., nitrate, phosphate, chloride, and ammonia) through soil and into local water sources. Microbes and chemicals usually traveled less than 15 meters from latrines, although some studies reported contamination up to about 25 meters away. Viruses were detected up to 50 meters from pit latrines. But different studies have found very different results even for the same location, reflecting a perplexing variety of experimental designs. Most groundwater contamination was reported downstream of pit latrines.

Pit latrines are an established, proven strategy for improving human waste disposal, but they can pose a tangible risk to drinking water resources in developing countries. The authors write that clear standards need to be established for siting pit latrines in relation to groundwater supplies. Future studies should also examine emerging contaminants and empirically test specific guidelines under a variety of conditions.

